# Small-angle neutron scattering modeling of spin disorder in nanoparticles

**DOI:** 10.1038/s41598-017-13457-2

**Published:** 2017-10-12

**Authors:** Laura G. Vivas, Rocio Yanes, Andreas Michels

**Affiliations:** 1 Physics and Materials Science Research Unit, University of Luxembourg, 162A avenue de la Faiencerie, Luxembourg, L-1511 Luxembourg; 2 Department of Applied Physics, University of Salamanca, Plaza de los Caidos, Salamanca, 37008 Spain

## Abstract

Magnetic small-angle neutron scattering (SANS) is a powerful technique for investigating magnetic nanoparticle assemblies in nonmagnetic matrices. For such microstructures, the standard theory of magnetic SANS assumes uniformly magnetized nanoparticles (macrospin model). However, there exist many experimental and theoretical studies which suggest that this assumption is violated: deviations from ellipsoidal particle shape, crystalline defects, or the interplay between various magnetic interactions (exchange, magnetic anisotropy, magnetostatics, external field) may lead to nonuniform spin structures. Therefore, a theoretical framework of magnetic SANS of nanoparticles needs to be developed. Here, we report numerical micromagnetic simulations of the static spin structure and related unpolarized magnetic SANS of a single cobalt nanorod. While in the saturated state the magnetic SANS cross section is (as expected) determined by the particle form factor, significant deviations appear for nonsaturated states; specifically, at remanence, domain-wall and vortex states emerge which result in a magnetic SANS signal that is composed of all three magnetization Fourier components, giving rise to a complex angular anisotropy on a two-dimensional detector. The strength of the micromagnetic simulation methodology is the possibility to decompose the cross section into the individual Fourier components, which allows one to draw important conclusions regarding the fundamentals of magnetic SANS.

## Introduction

Magnetic nanoparticles are the building blocks of the future magnetism-based technological applications^1,2^. At the nanoscale, complex spin structures such as vortices or skyrmions may appear^3^, which originate from the competition between various magnetic interactions and/or from geometrical constraints^4^. In order to image such spin structures, it is important to have observational techniques at hand which allow one to see the magnetization distribution on the nanometer length scale. Recent examples include spin-polarized scanning tunneling microscopy^3^, which has been employed to observe and manipulate skyrmions in ultra-thin films^5–7^, or electron holography, which has been used to investigate the temperature and magnetic-field dependence of the magnetic moments of individual skyrmions^8^, or to show that the structure of a vortex state can be adjusted by varying the aspect ratio of single-crystal hcp Co nanowires^9^. Small-angle neutron scattering (SANS) has also been recognized as a powerful technique for studying nanostructured magnetic materials; for instance, SANS is crucial for studying the skyrmion lattice in MnSi^10,11^, ferromagnetic nanorod and nanowire arrays^12–17^, magnetic nanoparticles^18–21^, bulk magnets including magnetic steels^22–26^, or the magnetic microstructure of nanocrystalline Nd-Fe-B magnets^27–29^. Indeed, due to the magnetic sensitivity and the high transparency of neutrons to matter, SANS provides nanometer-scale (~1–100 nm) information from within the bulk of a sample; these properties render SANS complementary to other observational experimental techniques which probe the local surface rather than the bulk structure.

**Figure 1:**
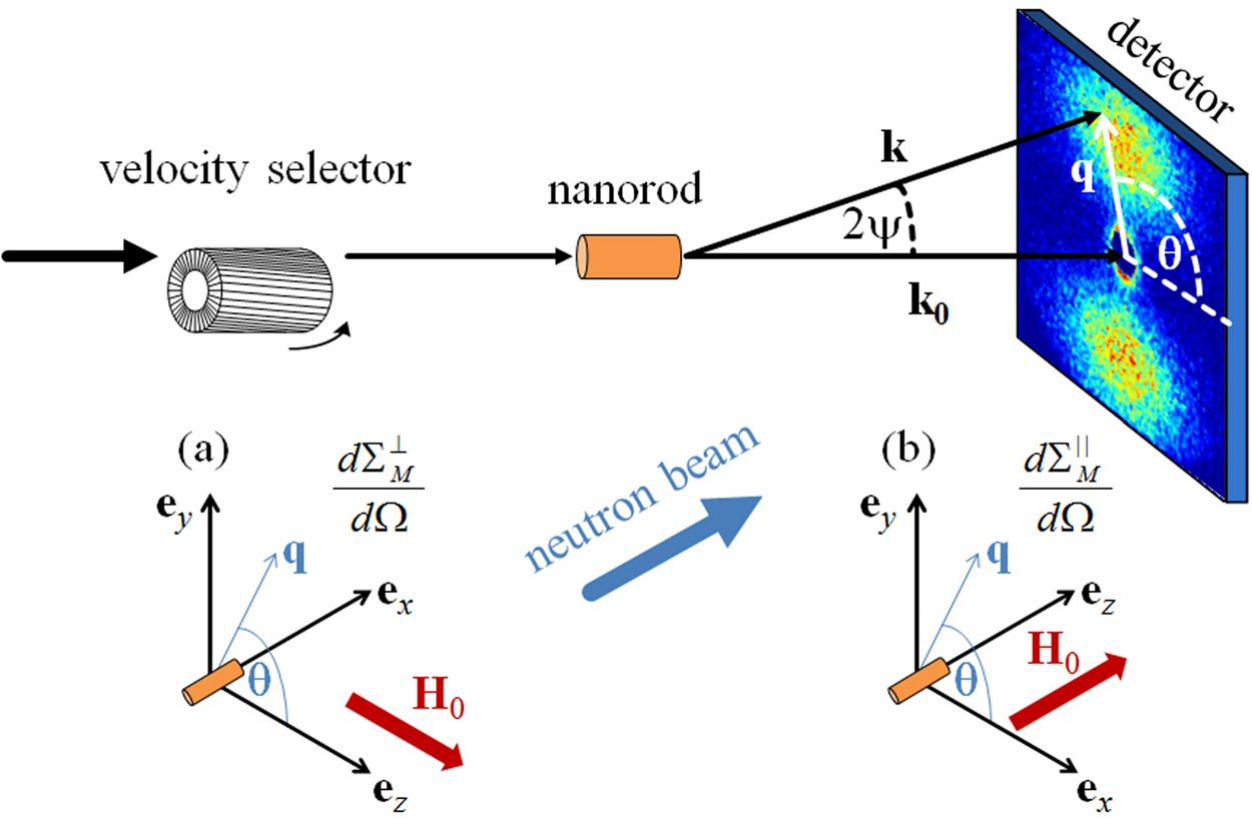
Sketch of the perpendicular (**a**) and parallel (**b**) SANS scattering geometries. Note that the applied magnetic field **H**_0_ is along ${{\bf{e}}}_{z}$ in both geometries and that the angle $\theta$ describes the orientation of the scattering vector **q** on the two-dimensional detector. The average neutron wavelength $\lambda$ is determined by the velocity selector; ${{\bf{k}}}_{0}$ and **k** denote the wave vectors of the incident and the scattered neutrons; $2\psi$ is the scattering angle, and $|{\bf{q}}|=q=\frac{4\pi }{\lambda }\,\sin \,\psi$. In the small-angle approximation, the component of **q** along the incident beam is ignored.

Whilst for bulk ferromagnets the theory of magnetic SANS has recently been developed^30–35^, for isolated magnetic nanoparticles in a nonmagnetic matrix the theoretical description of magnetic SANS is still in its infancy; in particular, when microstructural-defect or particle-shape-induced spin misalignment is present. In fact, the magnetic SANS theory of nanoparticles remains an open problem that needs to be resolved for an accurate analysis of experimental data. So far, magnetic SANS data are mostly analyzed by expressing the magnetic SANS cross section $d{{\rm{\Sigma }}}_{M}/d{\rm{\Omega }}$ at momentum-transfer or scattering vector **q** in terms of noninterfering single-particle form factors^19,36,37^,

1 $$\frac{d{{\rm{\Sigma }}}_{M}}{d{\rm{\Omega }}}({\bf{q}})=n{({\rm{\Delta }}{\rho }_{{\rm{mag}}})}^{2}{V}_{p}^{2}\,|F({\bf{q}}{)|}^{2}{\sin }^{{\rm{2}}}\alpha ,$$

where *n* is the number density of particles, ${\rm{\Delta }}{\rho }_{{\rm{mag}}}$ denotes the magnetic scattering-length density contrast between particle and matrix (essentially the difference in saturation magnetizations), *V*_*p*_ is the particle volume, *F*(**q**) its form factor, and the ${\sin }^{2}\alpha$ factor is due to the dipolar nature of the neutron-magnetic interaction. For a single-domain particle, *α* denotes the angle between **q** and the magnetization **M** of the particle^38^. Figure 1 displays sketches of the two most common (perpendicular and parallel) SANS scattering geometries.

For many problems the macrospin model that is embodied in equation (1) is oversimplified, since it assumes homogeneous (or stepwise homogeneous) magnetization and, thus, ignores the complex spin structures that may appear at the nanoscale. As mentioned above, intraparticle spin disorder is due to the interplay between different magnetic interactions such as surface anisotropy and dipolar interaction, or to the presence of crystal defects. Therefore, SANS approaches based on the “standard model” [equation (1)] might be erroneous when the spatial dependence of the *magnitude and direction* of the magnetization **M**(**r**) is not taken into account^31^. Günther *et al*.^16^ have suggested the presence of significant intraparticle spin disorder when analyzing SANS experiments on a Co nanorod array: the strong field dependence of $d{\rm{\Sigma }}/d{\rm{\Omega }}$ that these authors have observed could not be explained by the standard expression for $d{\rm{\Sigma }}/d{\rm{\Omega }}$. Likewise, the polarized SANS experiments on ferromagnetic nanowires by Napolskii *et al*.^14^ and by Maurer *et al*.^17^ indicate that the nanowires’ stray fields have to be taken into account in the magnetic form-factor derivation.

Micromagnetic computer simulations are ideally suited to tackle the above described problem, since one can include in a straightforward manner all the relevant inter- and intraparticle magnetic interactions (dipolar and Zeeman interactions, magnetic anisotropy, exchange), which in turn allows one to compute the magnetic SANS cross section for an inhomogeneously magnetized nanoparticle. Note that analytical micromagnetic calculations of the magnetic SANS cross section of nanoparticles are extremely difficult due to the nonlinear character of the underlying differential equations; a first attempt into this direction has been carried out by Metlov and Michels^34^, who computed the magnetic scattering related to vortices in thin submicron-sized soft ferromagnetic cylinders. Important aspects of computational micromagnetism are that (i) the parameter space can be relatively quickly scanned and that (ii) this methodology permits the investigation of the impact of the different interactions on magnetic SANS simply by switching on and off these interactions in simulations; such a procedure may then provide fundamental information on magnetic SANS. Up to now, however, this approach has only been used for studying bulk ferromagnets^39–42^, where not only the relevant magnetic interactions may strongly differ from those in nanoparticles, but where all phases (and not only the nanoparticle phase) comprising the material are ferromagnetic. Needless to say that for nanoparticles the particle shape also plays an important role for the spin configuration.

In this paper we employ micromagnetic computer simulations in order to investigate the magnetic SANS cross section of an isolated Co nanorod for different nanorod diameters. The choice of this particular system is motivated by the great interest from both the fundamental and technological point of view. Indeed, the structural and magnetic properties of elongated magnetic nanoobjects in the form of nanorods (or nanowires) have emerged as some of the most promising materials for functionalized magnetic nanostructures, with relevant interest in biomedicine^43^, permanent magnets^44^, and information technologies^4,45^. Cobalt nanowires may be suitable candidates for dense three-dimensional arrays of magnetic vortex-based media^9,46^ and for the integration in nanooscillators based on arrays of magnetostatically coupled Co/Au segmented nanopillars;^47^ in particular, synthesis improvements based on self-organization principles have made it possible to produce monocrystalline Co nanorods with controlled easy-axis orientations, which are frequently found to be almost perpendicular to the nanorod direction^46,48^. Depending on the orientation of the *c*-axis, the magnetization distribution at remanence can be tuned to be parallel to the nanowire axis, or to form a vortex structure when it is perpendicular.

The paper is organized as follows: we begin the discussion with the saturated SANS cross sections, where the problem is a purely geometrical one. We then continue to discuss the magnetization distributions and the ensuing magnetic SANS in the remanent state as a function of the diameter-to-length ratio; here, strongly inhomogeneous magnetization structures determine the SANS cross sections of the nanorod. The decomposition of $d{{\rm{\Sigma }}}_{M}/d{\rm{\Omega }}$ into the individual magnetization Fourier components in conjunction with computed hysteresis loops provides useful insights into the role of spin-misalignment scattering. The magnetic SANS cross sections for the two most often employed scattering geometries as well as information about the micromagnetic simulations can be found in the Methods section. We also refer the reader to the Supplemental Material, which contains many additional data.

## Results and Discussion

**Figure 2:**
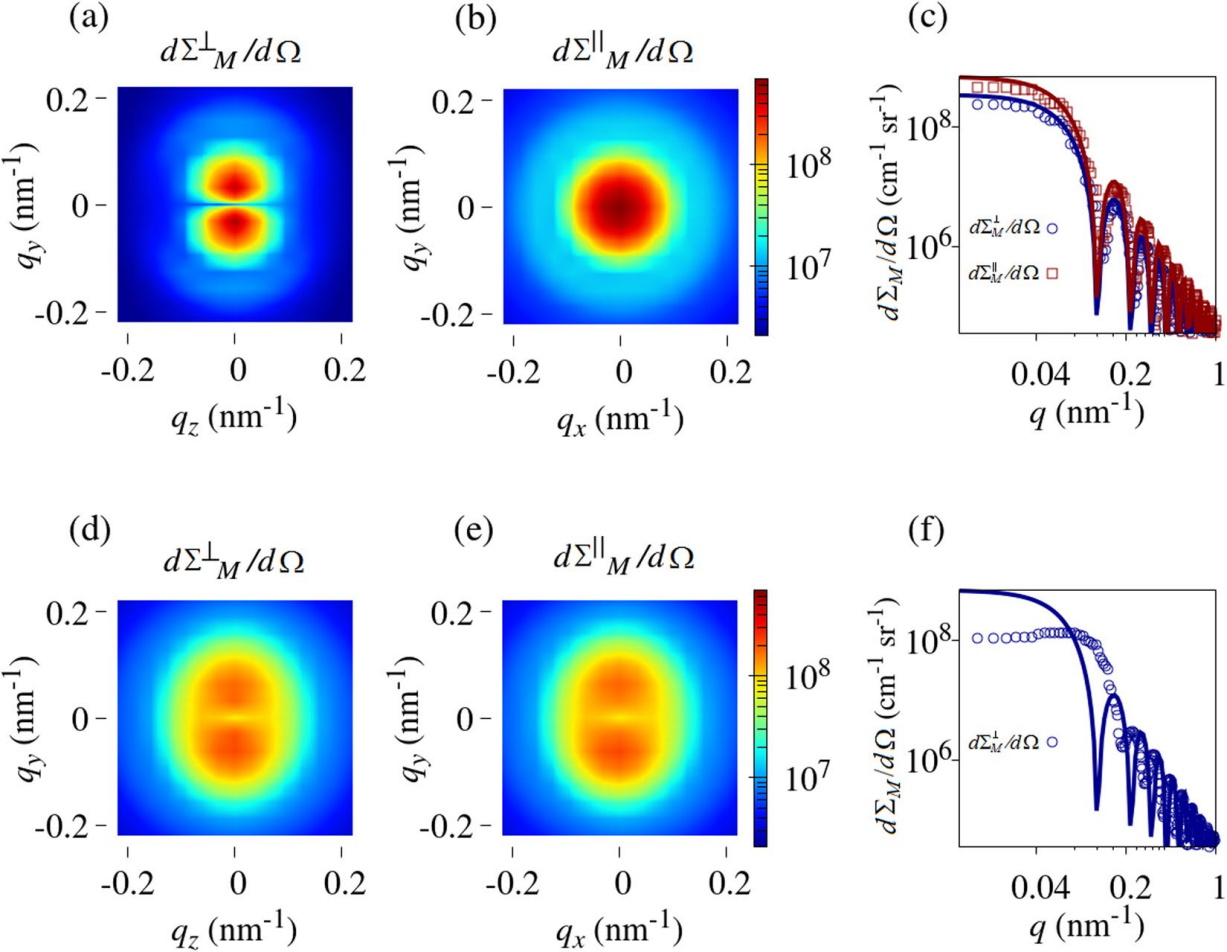
Computed magnetic SANS cross sections of a Co nanorod with a diameter of 60 nm and a length of 500 nm. (**a**)–(**c**) Saturated state; (**d**)–(**f**) remanent state $({H}_{0}=0)$. Shown are the two-dimensional $d{{\rm{\Sigma }}}_{M}/d{\rm{\Omega }}$ for the perpendicular (${{\bf{k}}}_{0}\perp {{\bf{H}}}_{0}$) and parallel $({{\bf{k}}}_{0}\,\parallel \,{{\bf{H}}}_{0})$ scattering geometries (logarithmic color scale) as well as the ($2\pi$) azimuthally-averaged $d{{\rm{\Sigma }}}_{M}/d{\rm{\Omega }}$. The solid lines in the azimuthally-averaged curves [(**c**) and (**f**)] represent the form factor of a cylinder which has its long axis oriented at an angle of 90° relative to the scattering vector [equations (1) and (2)] (log-log scale); note that in equation (1) ${\sin }^{2}\alpha =1/2$ (${{\bf{k}}}_{0}\perp {{\bf{H}}}_{0}$) and ${\sin }^{2}\alpha =1$ (${{\bf{k}}}_{0}\,\parallel \,{{\bf{H}}}_{0}$) at saturation, and ${\sin }^{2}\alpha =1$ in the remanent state for both geometries. For clarity, only $d{{\rm{\Sigma }}}_{M}^{\perp }/d{\rm{\Omega }}$ is shown in (**f**). **H**_0_ is horizontal in the plane in (**a**) and (**d**), and normal to the plane in (**b**) and (**e**).

In Fig. 2(a)-(c) we display the computed magnetic SANS cross section of a magnetically saturated Co nanorod with a length of $L=500\,{\rm{nm}}$ and a diameter of $D=60\,{\rm{nm}}$. At saturation, the magnetization state of the nanorod is given by ${\bf{M}}=\mathrm{(0,}\,\mathrm{0,}\,{M}_{z}={M}_{s})$, so that both $d{{\rm{\Sigma }}}_{M}^{\perp }/d{\rm{\Omega }}$ and $d{{\rm{\Sigma }}}_{M}^{\parallel }/d{\rm{\Omega }}$ [equations (4) and (5)] are exclusively determined by the Fourier coefficient ${\tilde{M}}_{z}$ of the longitudinal magnetization (see Methods section). By comparison to equations (4) and (5), it is then seen that the perpendicular SANS cross section exhibits a ${\sin }^{2}\theta$-type angular anisotropy, i.e., $d{{\rm{\Sigma }}}_{M}^{\perp }/d{\rm{\Omega }}$ is elongated normal to **H**_0_, while the SANS cross section in the parallel scattering geometry is isotropic. The azimuthally-averaged data $[{\mathrm{(2}\pi )}^{-1}{\int }_{0}^{2\pi }\mathrm{(...)}d\theta ]$ for the perpendicular case (and likewise for ${{\bf{k}}}_{0}\,\parallel \,{{\bf{H}}}_{0}$) can be very well described by the form factor of a circular disc,

2 $${F}^{2}(q)={(\frac{2{J}_{1}(qR)}{qR})}^{2},$$

where ${J}_{1}(z)$ denotes the first-order Bessel function and *R* is the nanorod’s radius [solid lines in Fig. 2(c)]; in fact, $d{{\rm{\Sigma }}}_{M}^{\perp }/d{\rm{\Omega }}$ and $d{{\rm{\Sigma }}}_{M}^{\parallel }/d{\rm{\Omega }}$ are identical at saturation, except for a factor of 1/2 which comes in on azimuthally averaging equation (4). The results depicted in Fig. 2(a)–(c) confirm the expected behavior for which analytical results exist.

**Figure 3:**
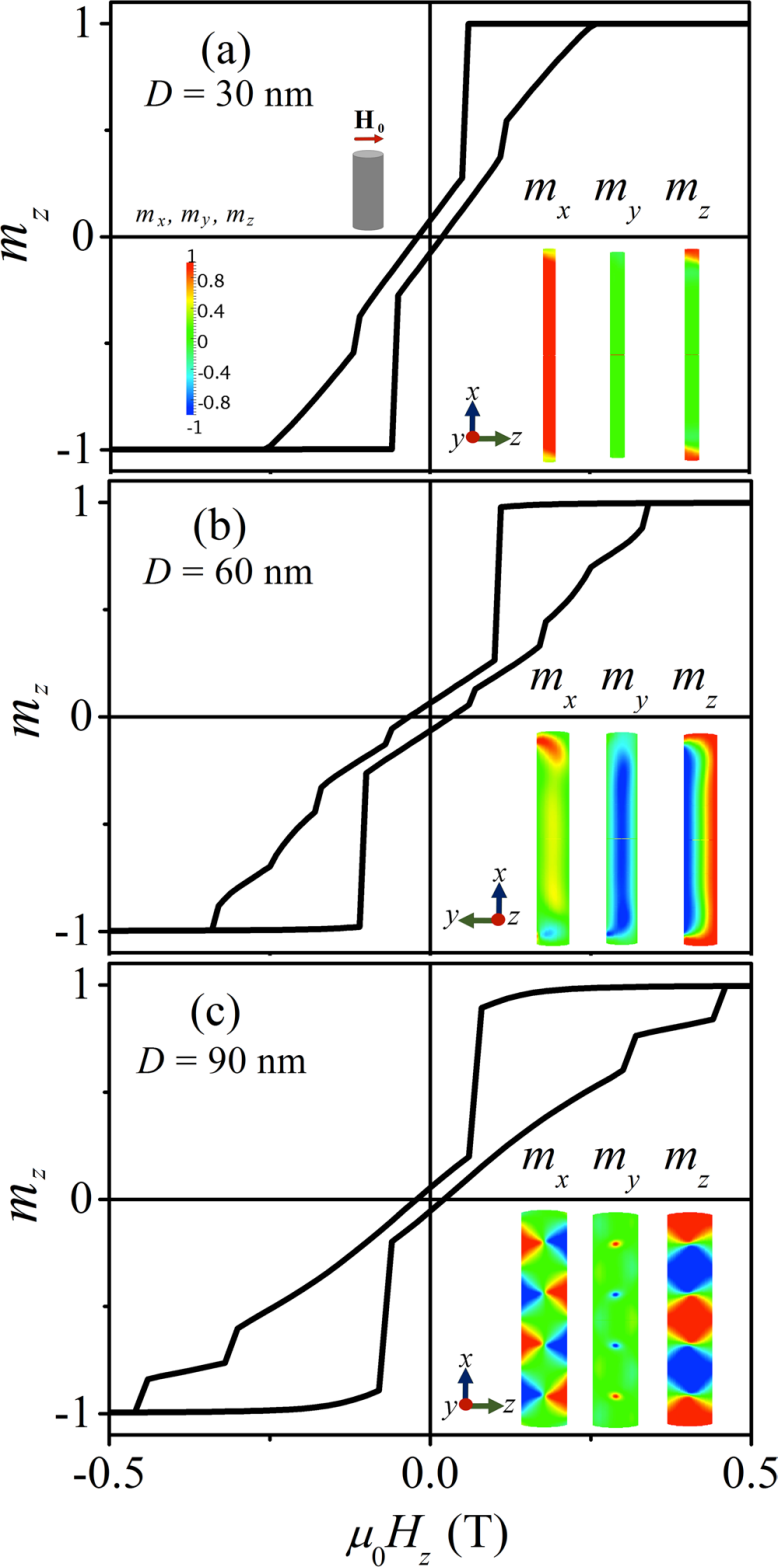
Computed hysteresis loops of Co nanowires with a length of $L=500\,{\rm{nm}}$ and diameters of (**a**) $D=30\,{\rm{nm}}$, (**b**) $D=60\,{\rm{nm}}$, and (**c**) $D=90\,{\rm{nm}}$. The uniaxial magnetocrystalline anisotropy axis points along the $\pm z$-direction. The insets depict color-coded representations of the spin structure in the remanent state. Note that in (**b**) the coordinate axes have been rotated by 90° around the *x*-axis.

In the following we discuss the spin structures and related magnetic SANS cross sections in the remanent state as a function of the *D*/*L* ratio; the length of the nanorod is fixed at $L=500\,{\rm{nm}}$ and the diameter is varied between $D=\mathrm{30,60}$, and $90\,{\rm{nm}}$. As is well known, for such shape-anisotropic particles, the long-range magnetodipolar interaction gives rise to magnetic shape anisotropy, which tends to align the magnetic moments along the nanorod’s long axis. On top of that, we consider an uniaxial magnetocrystalline anisotropy axis ${{\bf{K}}}_{u}$, which is directed along the cylinder’s diameter (perpendicular to the long axis). As a consequence, the system behaves effectively like a biaxial magnetic material and the spin structure reflects the competition between both interactions; in particular, as *D* increases at constant *L* and at constant magnitude and direction of ${{\bf{K}}}_{u}$. Magnetization curves and spin structures for ${K}_{u}=0$ are shown in the Supplemental Material (Figs. 1 and 3).

Figure 2(d)-(f) depict the perpendicular and parallel $d{{\rm{\Sigma }}}_{M}/d{\rm{\Omega }}$ at remanence $({H}_{0}=0)$, where the rod’s long axis for both scattering geometries is parallel to the normal on the detector plane. For an inhomogeneously magnetized nanorod, the magnetization components ${M}_{x,y,z}$ depend explicitly on the position ${\bf{r}}=\{x,y,z\}$ and all three Fourier components ${\tilde{M}}_{x,y,z}$ contribute to $d{{\rm{\Sigma }}}_{M}/d{\rm{\Omega }}$. It is seen in Fig. 2(d)-(e) that a more complicated SANS pattern than in the saturated state results (see detailed discussion below); we also note that although both $d{{\rm{\Sigma }}}_{M}/d{\rm{\Omega }}$ are nearly identical, the corresponding magnetization curves (for **H**_0_ perpendicular and parallel to the long axis) are quite different (compare Fig. 3(b) in the paper with Fig. 2(b) in the Supplemental Material). Importantly, the azimuthally-averaged data in Fig. 2(f) cannot be described anymore by the geometrical form-factor model [equations (1) and (2)], which assumes a uniform magnetization state. Away from saturation, the magnetic microstructure decomposes into several domains, which naturally have an average spatial extension that is *smaller* than the purely geometrical dimensions (*L*, *D*) of the nanorod. Therefore, the “form-factor” oscillations corresponding to these domains appear at *larger* values of the momentum transfer, as it becomes visible in Fig. 2(f). Note that in the following we restrict our considerations to the case of ${{\bf{k}}}_{0}\perp {{\bf{H}}}_{0}$, which is the most often employed scattering geometry in magnetic SANS experiments.

Before we discuss in more detail the magnetic scattering cross sections of the nanowire, we would like to show in Fig. 3 some results for the hysteresis curves and internal spin structures at remanence; note that the applied field in Fig. 3 is directed along the ${{\bf{K}}}_{u}$-axis. For $D=30\,{\rm{nm}}$ and a corresponding relatively small ratio of $D/L=0.06$ [Fig. 3(a)], the spin structures are determined by the magnetic shape anisotropy (compare also Fig. 2(a) and left part in Fig. 4 in the Supplemental Material). When ${H}_{0}={H}_{z}$ is reduced starting from saturation, the magnetization will rotate towards the shape-anisotropy axis. At remanence, the magnetization is then predominantly oriented along the *x*-direction, with some minor spin misalignment along the *z*-direction at the nanowire’s end faces, and a vanishingly small magnetization component along the *y*-direction. The localized spin inhomogeneities at the end faces are related to the combined effects of the inhomogeneous dipole field of the rod, which tends to align the moments parallel to the surface of the nanorod’s ends, the exchange interaction, which tries to keep the ferromagnetic order, and the magnetocrystalline anisotropy, which prefers the magnetization along the ±*z*-direction. In the center part of the $D=30\,{\rm{nm}}$ nanorod, the magnetic state at remanence is quite similar to the case without magnetocrystalline anisotropy (see Fig. 1(a) and upper left part in Fig. 3 in the Supplemental Material); the main changes between the hysteresis loops with and without magnetocrystalline anisotropy are the reduction of the field that is needed to saturate the system along the *z*-direction and the existence of a small coercivity. We will see below that as *D* increases (at constant *L* and ${K}_{u}$), the (reduced) dipolar interaction favors more complex magnetic structures such as domain wall or vortex states, which minimize the magnetic flux through the surface of the system.

**Figure 4:**
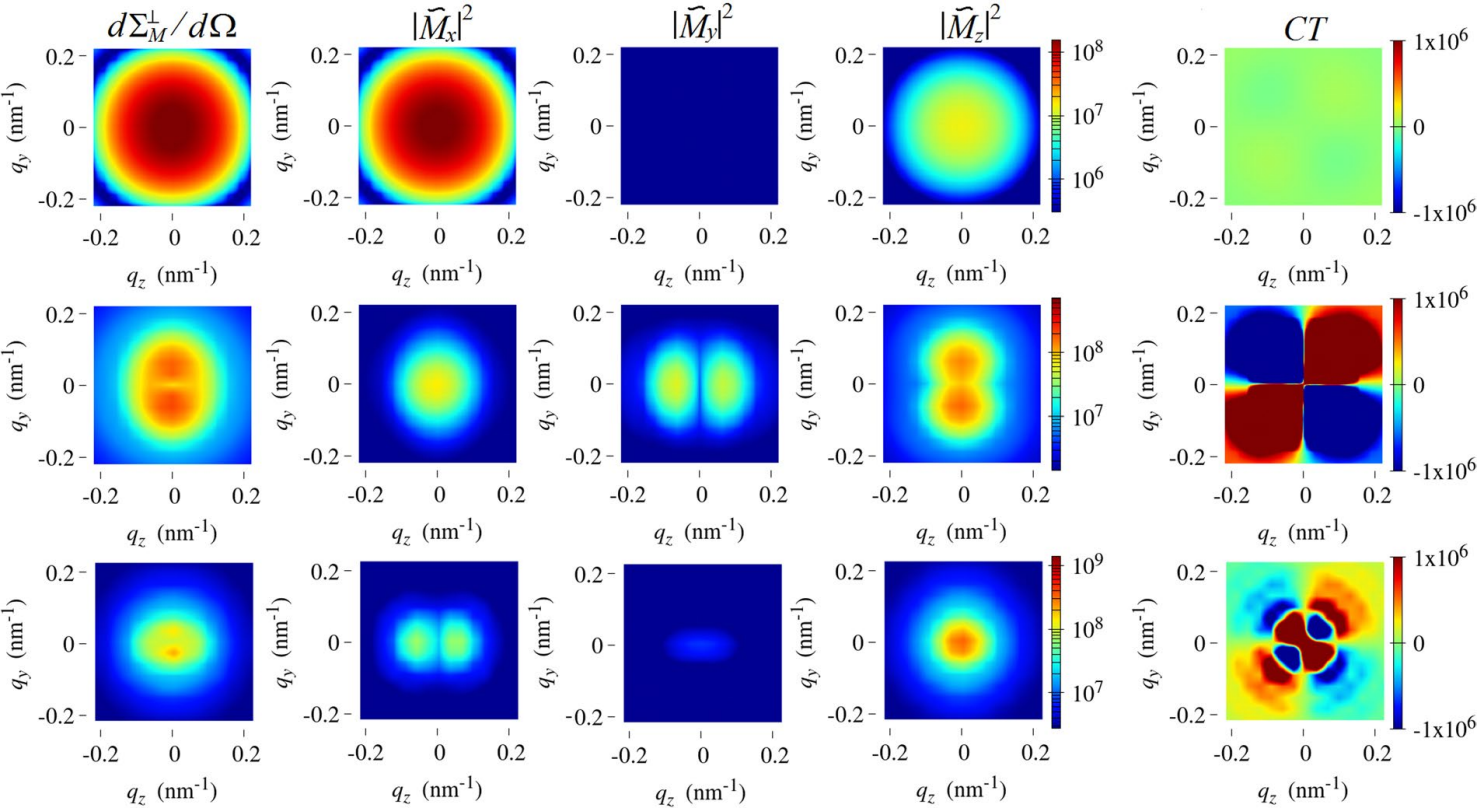
Results of the micromagnetic simulations for the total magnetic SANS cross section and the Fourier components of the magnetization in the remanent state (${{\bf{k}}}_{0}\perp {{\bf{H}}}_{0}$). The images represent projections of $d{{\rm{\Sigma }}}_{M}^{\perp }/d{\rm{\Omega }}$, $|\tilde{{M}_{x}}{|}^{2}$, $|\tilde{{M}_{y}}{|}^{2}$, $|\tilde{{M}_{z}}{|}^{2}$ (all on a logarithmic color scale) and of the cross term $CT=-({\tilde{M}}_{y}^{\ast }\tilde{{M}_{z}}+{\tilde{M}}_{z}^{\ast }\tilde{{M}_{y}})$ (linear color scale) into the plane of the two-dimensional detector (${q}_{x}=0$). **H**_0_ is horizontal in the plane. $D=30\,{\rm{nm}}$ (upper row); $D=60\,{\rm{nm}}$ (middle row); $D=90\,{\rm{nm}}$ (lower row). Compare to Fig. 6 in the Supplemental Material, which depicts the corresponding results for ${{\bf{k}}}_{0}\,\parallel \,{{\bf{H}}}_{0}$.

Based on these (real-space) observations, the corresponding (reciprocal-space) SANS cross section and the magnetization Fourier components for the $D=30\,{\rm{nm}}$ nanorod (upper row in Fig. 4) can be understood: $d{{\rm{\Sigma }}}_{M}^{\perp }/d{\rm{\Omega }}$ is dominated by the $|\tilde{{M}_{x}}{|}^{2}$ Fourier component and contains a weak $|\tilde{{M}_{z}}{|}^{2}$ contribution; the cross term $CT=-(\tilde{{M}_{y}^{\ast }}\tilde{{M}_{z}}+\tilde{{M}_{z}^{\ast }}\tilde{{M}_{y}})$ is negligible, since ${\tilde{M}}_{y}$ becomes very small. We would like to emphasize that the latter combination of Fourier components takes on positive as well as negative values (compare scale). However, on multiplication with the trigonometric function $\sin \,\theta \,\cos \,\theta$ in order to obtain the corresponding contribution to $d{{\rm{\Sigma }}}_{M}^{\perp }/d{\rm{\Omega }}$ [compare equation (4)] this term becomes positive-definite, since $\sin \,\theta \,\cos \,\theta$ changes sign at the borders between quadrants on the detector (positive for ${0}^{\circ } < \theta  < {90}^{\circ }$, negative for ${90}^{\circ } < \theta  < {180}^{\circ }$, and so on).

**Figure 5:**
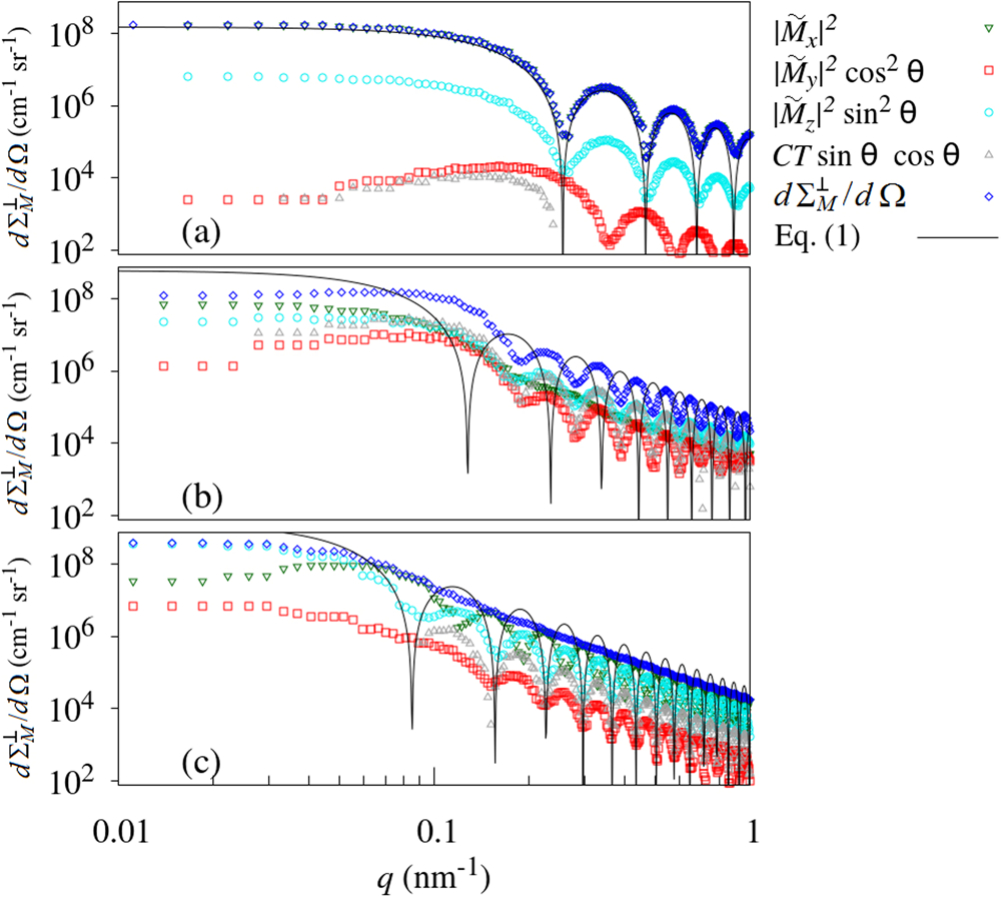
$2\pi$-azimuthally-averaged remanent-state data from Fig. 4 (see inset) (${{\bf{k}}}_{0}\perp {{\bf{H}}}_{0}$) (log-log scale). (**a**) $D=30\,{\rm{nm}}$; (**b**) $D=60\,{\rm{nm}}$; (**c**) $D=90\,{\rm{nm}}$. Solid lines in (**a**)–(**c**): equation (1) using the cicular-disc form factor of equation (2) and ${\sin }^{2}\alpha =1$.

Figure 5 depicts the ($2\pi$) azimuthal averages of $d{{\rm{\Sigma }}}_{M}^{\perp }/d{\rm{\Omega }}$ and of the individual Fourier components in the remanent state; this representation decrypts the magnetic SANS cross section and highlights the magnitudes and *q*-dependencies of the different scattering contributions. The solid lines in Fig. 5 are the prediction by equation (1) using the cicular-disc form factor [equation (2)] and a value of 1 for the expectation value of the ${\sin }^{2}\alpha$ factor, as appropriate for the zero-field spin configuration of a homogeneously magnetized nanorod particle (compare discussion related to equation (3) below). It is seen that only the $d{{\rm{\Sigma }}}_{M}^{\perp }/d{\rm{\Omega }}$ of the $D=30\,{\rm{nm}}$ nanorod can be well described by this function. The pronounced form-factor oscillations in $d{{\rm{\Sigma }}}_{M}^{\perp }/d{\rm{\Omega }}$ are progressively washed out with increasing diameter. Asymptotically, all data sets show $d{{\rm{\Sigma }}}_{M}^{\perp }/d{\rm{\Omega }}\propto {q}^{-3}$, in agreement with equation (2). It is also worth noting that the positions of the minima and maxima in $d{{\rm{\Sigma }}}_{M}^{\perp }/d{\rm{\Omega }}$ and in the Fourier components contain information about the characteristic size of magnetic domain features. However, in the absence of analytical theory for the Fourier components and in view of the facts that (i) the shape of the domains is field-dependent and not spherical (no single size parameter) and (ii) there exist no sharp boundaries between domains, the detailed analysis of the field dependence of the Fourier components and their relation to the real-space magnetization distribution is beyond the scope of this paper.

As mentioned above, increasing the nanorod’s diameter results in the emergence of a variety of magnetization switching processes and complex inhomogeneous states, in agreement with results by other authors^48–51^; this is in contrast to the case of vanishing magnetocrystalline anisotropy (see Figs. 1 and 3 in the Supplemental Material). While for $D=30\,{\rm{nm}}$ the shape anisotropy determines the magnetic configuration at remanence [Fig. 3(a)], changing the diameter to $D=60\,{\rm{nm}}$ ($D/L=0.12$) [Fig. 3(b)] reduces the strength of the shape anisotropy and the competition with the magnetocrystalline anisotropy leads to a change in the switching mechanism from essentially coherent rotation to the nucleation and propagation of a transversal domain wall. The domain oriented along the *x*-direction grows by propagating a transversal domain wall at the lateral boundaries of the cylinder [compare inset in Fig. 3(b)]. Therefore, the remanent state is characterized by this transversal domain wall. The related SANS cross section (middle row in Fig. 4) can be seen as a combination of all three magnetization Fourier components; note also that the *CT* term is now increased in magnitude as compared to the $D=30\,{\rm{nm}}$ case (due to an increased ${\tilde{M}}_{y}$ contribution). Notably, the circular symmetry of the magnetic SANS cross section observed at remanence for the smallest diameter has now turned into a (slightly) vertically elongated pattern due to the existence of magnetization components perpendicular to the rod’s axis, which directly result from the tranversal domain-wall configuration.

**Figure 6:**
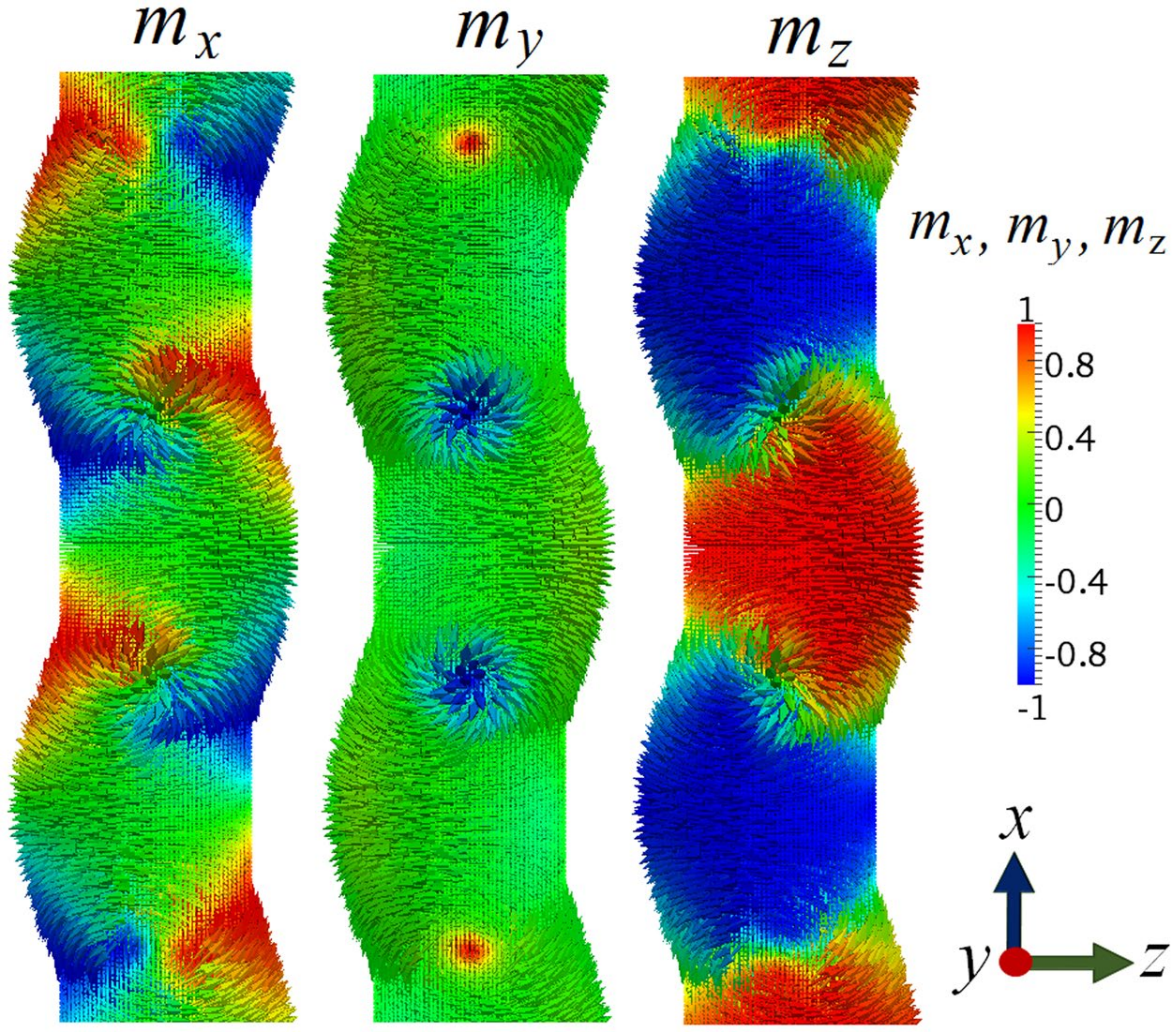
Computed real-space spin structure of a $D=90\,{\rm{nm}}$ nanorod at the remament state. The magnetization distributions are identical in all three images; the color scale encodes the different Cartesian components of **m** [compare inset in Fig. 3(c)]. The wavy-like modulation of the nanorods’s shape is an artifact related to representation of the spatial dependence of the magnetization vector field via arrows.

Finally, when the diameter is increased to $D=90\,{\rm{nm}}$ corresponding to an aspect ratio of $D/L=0.18$ [Fig. 3(c)], there is again a change in the mechanism from the nucleation and propagation of transversal domain-wall-like structures to vortex-like configurations. Indeed, the spin texture which stabilizes the system at remanence is a combination of different vortices (in those structures the magnetization is confined in the *x*-*z*-plane) with their cores aligned parallel and antiparallel to the *y*-direction, in other words, the vortex cores are oriented perpendicular to the plane that is spanned by the uniaxial and shape-anisotropy axes. As an example, Fig. 6 shows the spin distribution of the $D=90\,{\rm{nm}}$ nanorod. Consequently, the $|{\tilde{M}}_{y}{|}^{2}$ Fourier component is small in magnitude and the SANS cross section (lower row in Fig. 4) is dominated by $|{\tilde{M}}_{x}{|}^{2}$ and $|{\tilde{M}}_{z}{|}^{2}$, resulting in a distorted pattern with a slight elongation along the field direction. By comparison of the magnetization Fourier components along the rod (*x*) and the magnetocrystalline (*z*) axes, it can be seen that the shape anisotropy still contributes to the effective anisotropy, however, its strength is lowered as compared to the anisotropy created parallel to the rod’s diameter, i.e., increasing the diameter reinforces the effect of the magnetocrystalline anisotropy. The *CT* term contributes to $d{{\rm{\Sigma }}}_{M}^{\perp }/d{\rm{\Omega }}$ only with a low signal, which is related to the small volume fraction of vortex cores (small ${\tilde{M}}_{y}$ contribution, compare middle image in Fig. 6). Interestingly, at the smallest *q*, the *CT* term has inverted its sign compared to the $D=30\,{\rm{nm}}$ and $D=60\,{\rm{nm}}$ results, which may reflect an asymmetry of the magnetic configuration. In monocrystalline hcp Co nanowire arrays with $D/L=0.225$, Ivanov *et al*.^9,46^ have demonstrated that, at remanence, the magnetic structure can be composed of multiple stable magnetic vortex domains with different chirality.

The data for the individual Fourier components in Fig. 4 also reveal that these functions may explicitly depend on the angle $\theta$ in the detector plane. For bulk ferromagnets, it has been demonstrated by means of micromagnetic simulations that such an angular anisotropy is related to the magnetodipolar interaction^39–42^. We emphasize that the possible $\theta$-dependency of the Fourier components adds on top of the trigonometric functions in the magnetic SANS cross sections [equations (4) and (5)], which are due to the dipolar nature of the neutron-magnetic matter interaction.

**Figure 7:**
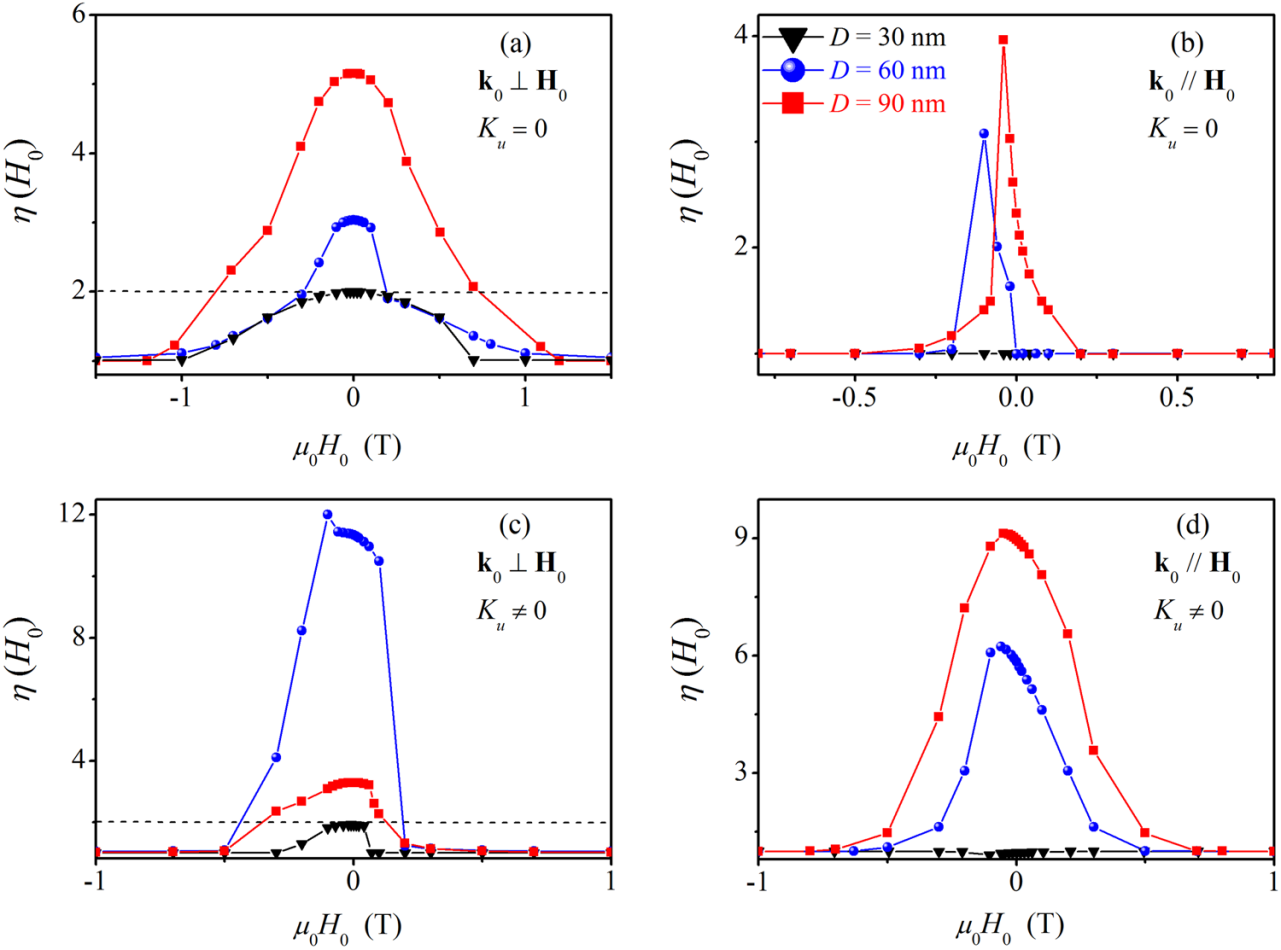
The function $\eta ({H}_{0})$ [equation (3)] computed for several nanorod diameters *D* [see inset in (b)] (lines are guides to the eyes). (**a**) ${{\bf{k}}}_{0}\perp {{\bf{H}}}_{0}$ and zero magnetocrystalline anisotropy $({K}_{u}=0)$; (**b**) ${{\bf{k}}}_{0}\,\parallel \,{{\bf{H}}}_{0}$ and ${K}_{u}=0$; (**c**) ${{\bf{k}}}_{0}\perp {{\bf{H}}}_{0}$ and ${K}_{u}=4.5\times {10}^{5}\,{\rm{J}}/{{\rm{m}}}^{{\rm{3}}}$; (**d**) ${{\bf{k}}}_{0}\,\parallel \,{{\bf{H}}}_{0}$ and ${K}_{u}=4.5\times {10}^{5}\,{\rm{J}}/{{\rm{m}}}^{{\rm{3}}}$. Dashed lines in (**a**) and (**c**): Stoner-Wohlfarth limit.

In order to grasp the deviation between the standard model [equation (1)] and the spin-misalignment SANS into one single parameter, we introduce the function

3 $$\eta ({H}_{0})=\frac{d{{\rm{\Sigma }}}_{M}}{d{\rm{\Omega }}}({q}^{\ast },{H}_{0})/\frac{d{{\rm{\Sigma }}}_{M}}{d{\rm{\Omega }}}({q}^{\ast },{H}_{0}\to \infty ),$$

which describes the normalized variation of the azimuthally-averaged magnetic SANS cross section $d{{\rm{\Sigma }}}_{M}/d{\rm{\Omega }}$ with the applied magnetic field. Figure 7 shows the results for $\eta ({H}_{0})$ obtained by computing $d{{\rm{\Sigma }}}_{M}/d{\rm{\Omega }}$ of the Co nanorod for both scattering geometries and for several *H*_0_-values between positive and negative saturation with and without magnetocrystalline anisotropy. For the computation of $\eta$, the average intensity at the respective characteristic *q*-value of ${q}^{\ast }=2\pi /D$ was used. The behavior of $\eta ({H}_{0})$ for a *uniformly* magnetized shape-anisotropic nanoparticle [cf. the discussion regarding equation (1)] and for the two scattering geometries can be understood within the context of the Stoner-Wohlfarth model^52^: for ${{\bf{k}}}_{0}\parallel {{\bf{H}}}_{0}$ (corresponding to the case that the rod’s long axis is parallel to **H**_0_), the magnetization curve exhibits the well-known rectangular shape, implying that **M** points always along the *z*-direction; there is, of course, a switching from the +*z* to the −*z* direction at the nucleation field, but this feature appears to be too “sharp” to be resolved with the SANS technique. As a consequence, the scattering vector **q** is always perpendicular to **M**, so that the expectation value of the ${\sin }^{2}\alpha$ factor in equation (1) equals unity and the function $\eta ({H}_{0})$ does *not* depend on the field; the corresponding scattering pattern is isotropic. For ${{\bf{k}}}_{0}\perp {{\bf{H}}}_{0}$ (corresponding to the case that the rod’s long axis is perpendicular to **H**_0_), the Stoner-Wohlfarth model predicts a linear paramagnetic-like decrease from saturation (along the horizontal *z*-direction) to a zero remanent magnetization, where now (due to the shape anisotropy) the magnetization points along the wire axis (*x*-direction). The expectation value of ${\sin }^{2}\alpha$ changes from a value of 1/2 at saturation to a value of 1 at ${H}_{0}=0$, in other words, $\eta ({H}_{0})$ increases from 1 to a value of 2.

The above described idealized Stoner-Wohlfarth scenario is fairly well reproduced by our simulations for the smallest diameter of $D=30\,{\rm{nm}}$ [black curves in Fig. 7; compare also Fig. 5(a)]; the very small deviations from $\eta =2$ in the perpendicular geometry [${K}_{u}\ne 0$; Fig. 7(c)] and from $\eta =1$ in the parallel geometry [${K}_{u}\ne 0$; Fig. 7(d)] are attributed to the small spin-misalignment scattering that is related to the spin disorder at the end faces of the cylinder [compare insets in Fig. 3(a)]. For the two largest diameters (60 nm and 90 nm), we observe a significant deviation from the Stoner-Wohlfarth prediction [and therefore also from the standard model, equation (1)] due to the emerging spin-disorder scattering [blue and red curves in Fig. 7; compare Fig. 5b,c]. While for both ${K}_{u}=0$ cases and for ${K}_{u}\ne 0$ in the parallel geometry we observe the trend that the spin-inhomogeneity parameter increases with increasing $D/L$, the behavior of $\eta ({H}_{0})$ is different for ${K}_{u}\ne 0$ in the perpendicular geometry [Fig. 7(c)]; here, the $D=90\,{\rm{nm}}$ rod exhibts a variation which is much smaller than the one of the $D=60\,{\rm{nm}}$ cylinder. This observation indicates that the magnitude of the spin-misalignment scattering of nanorods depends sensitively on their domain structure, which is determined by the nanoparticle’s geometry (ratio *D*/*L*) and by the magnetic interactions, including the applied-field direction (compare also the Fourier images in Fig. 4). All in all, the results depicted in Fig. 7 reveal–for not too small nanoparticles–significant deviations from the standard model due to internal spin disorder. In order to account for these inhomogeneous spin structures and to understand the associated complex scattering patterns, micromagnetic simulations provide the key tool set for progressing the understanding of magnetic SANS on nanoparticles.

## Conclusion and Outlook

We have presented the results of micromagnetic simulations of the magnetic microstructure and the ensuing magnetic small-angle neutron scattering (SANS) cross section of a single Co nanorod with a length of $L=500\,{\rm{nm}}$ and diameters of $D=\mathrm{30,}\,60$ and 90 nm. This methodology has allowed us to unravel the intrinsic contribution of the spin misalignment to the total magnetic SANS cross section via the study of the individual magnetization Fourier components; it provides important fundamental information regarding the explicit **q**-dependence of $d{{\rm{\Sigma }}}_{M}/d{\rm{\Omega }}$ as a function of the applied magnetic field, the nanoparticle’s microstructure, and the magnetic interactions. We anticipate that our investigation represents a starting point towards the development of a framework for computer-simulations-supported SANS-data analyses of magnetic nanoparticle assemblies exhibiting strong internal spin misalignment; for such systems, the classical geometrical description of magnetic SANS in terms of particle form factors is not appropriate (as embodied e.g. in Fig. 7), since in this approach the underlying magnetic problem is not addressed. Although the continuum theory of micromagnetics has been used in this paper, the framework can be easily extended to atomistic spin models^53^. Likewise, future simulation work–besides studying other particle shapes such as ellipsoids–will concentrate on (i) the scrutiny of the periodicity of the Fourier coefficients and their relation to the real-space spin structure, (ii) the study of interparticle interactions, which will then allow for a comparison of simulation results to experimental data, (iii) the inclusion of random variations of the magnetic anisotropy field in order to better describe microstructural defects (polycrystallinity), (iv) the inclusion of thermal fluctuations, and (v) the study of the magnetization dynamics by solving the Landau-Lifshitz-Gilbert equation.

## Methods

### Magnetic SANS cross section

The quantity of interest in our micromagnetic study is the elastic magnetic differential scattering cross section $d{{\rm{\Sigma }}}_{M}/d{\rm{\Omega }}$, which is usually recorded on a two-dimensional position-sensitive detector. For the most commonly used scattering geometries in magnetic SANS experiments (see Fig. 1), where the applied magnetic field ${{\bf{H}}}_{0}\,\parallel \,{{\bf{e}}}_{z}$ is either perpendicular (${{\bf{k}}}_{0}\perp {{\bf{H}}}_{0}$) or parallel (${{\bf{k}}}_{0}\,\parallel \,{{\bf{H}}}_{0}$) to the wave vector **k**_0_ of the incident neutrons, $d{{\rm{\Sigma }}}_{M}/d{\rm{\Omega }}$ for unpolarized neutrons can be written as^31^:

4 $$\frac{d{{\rm{\Sigma }}}_{M}^{\perp }}{d{\rm{\Omega }}}({\bf{q}})=\frac{8{\pi }^{3}}{V}{b}_{H}^{2}[|\tilde{{M}_{x}}{|}^{2}+|\tilde{{M}_{y}}{|}^{2}{\cos }^{2}\theta +|\tilde{{M}_{z}}{|}^{2}{\sin }^{2}\theta -(\tilde{{M}_{y}^{\ast }}\tilde{{M}_{z}}+\tilde{{M}_{z}^{\ast }}\tilde{{M}_{y}})\sin \,\theta \,\cos \,\theta ],$$

5 $$\frac{d{{\rm{\Sigma }}}_{M}^{\parallel }}{d{\rm{\Omega }}}({\bf{q}})=\frac{8{\pi }^{3}}{V}{b}_{H}^{2}[|{\tilde{{M}}}_{x}{|}^{2}{\sin }^{2}\theta +|{\tilde{{M}}}_{y}{|}^{2}{\cos }^{2}\theta +|{\tilde{{M}}}_{z}{|}^{2}-(\tilde{{M}_{x}^{\ast }}\tilde{{M}_{y}}+\tilde{{M}_{y}^{\ast }}\tilde{{M}_{x}})\sin \,\theta \,\cos \,\theta ]\mathrm{.}$$

In equations (4) and (5), *V* denotes the scattering volume ($\pi {R}^{2}L$ in our study), ${b}_{H}=2.70\times {10}^{-15}m/{\mu }_{B}$ with ${\mu }_{B}$ the Bohr magneton, and $\mathop{{\bf{M}}}\limits^{ \sim }({\bf{q}})=\{{\mathop{M}\limits^{ \sim }}_{x}({\bf{q}}),{\mathop{M}\limits^{ \sim }}_{y}({\bf{q}}),{\mathop{M}\limits^{ \sim }}_{z}({\bf{q}})\}$ represents the Fourier transform of the magnetization vector field ${\bf{M}}({\bf{r}})=\{{M}_{x}({\bf{r}}),{M}_{y}({\bf{r}}),{M}_{z}({\bf{r}})\}$; the asterisks “*” mark the complex-conjugated quantity. Note that in the small-angle limit the momentum-transfer vector is given by ${\bf{q}}\cong q\mathrm{(0,}\,\sin \,\theta ,\,\cos \,\theta )$ for ${{\bf{k}}}_{0}\perp {{\bf{H}}}_{0}$, where $\theta$ denotes the angle between **q** and **H**_0_, whereas $q\cong q(\cos \,\theta ,\,\sin \,\theta \mathrm{,\; 0)}$ for ${{\bf{k}}}_{0}\,\parallel \,{{\bf{H}}}_{0}$, where $\theta$ is measured between **q** and **e**_*x*_. These expressions demonstrate that SANS mainly probes correlations in the plane normal to **k**_0_. The applied field **H**_0_ defines the *z*-axis of a Cartesian laboratory coordinate system for both geometries. Since the focus of this study is on magnetic (spin-misalignment) scattering, we have ignored the nuclear SANS contribution, which for a homogeneous particle can be described by the particle form factor. The magnetization Fourier amplitudes depend on the size and shape of the particle, but most importantly on the magnetic interactions determining the nanoparticle’s spin structure. Using computer simulations based on the continuum theory of micromagnetics it becomes possible to include all the relevant intraparticle magnetic interactions such as magnetic anisotropy, magnetostatics, and exchange interaction. For a saturated nanoparticle, expressions (4) and (5) reduce to equations of the form of equation (1). With the aid of polarized neutrons it becomes possible to access nuclear-magnetic interference terms as well as purely chiral magnetic contributions.

### Micromagnetic simulations

We have performed micromagnetic simulations using the GPU-based open-source software package MuMax3^54^, which can calculate the space and time-dependent magnetic microstructure of nano- and micron-sized ferromagnets. Mumax3 employs a finite-difference discretization scheme of space using a two-dimensional or three-dimensional grid of orthorhombic cells. In the micromagnetic simulations we have taken into account all four standard contributions to the total magnetic Gibbs free energy: energy in the external magnetic field ${ {\mathcal E} }_{Z}$, magnetodipolar interaction energy ${ {\mathcal E} }_{D}$, energy of the (uniaxial) magnetocrystalline anisotropy ${ {\mathcal E} }_{{\rm{ani}}}$, and isotropic and symmetric exchange energy ${ {\mathcal E} }_{{\rm{ex}}}$:

6 $${ {\mathcal E} }_{Z}=-{\mu }_{0}{\int }_{V}{\bf{M}}\cdot {{\bf{H}}}_{0}\,dV,\quad { {\mathcal E} }_{D}=-\frac{1}{2}{\mu }_{0}{\int }_{V}{\bf{M}}\cdot {{\bf{H}}}_{D}dV,\quad { {\mathcal E} }_{{\rm{ani}}}=-{\int }_{V}{K}_{u}{({\bf{m}}\cdot {\bf{u}})}^{2}dV,\quad { {\mathcal E} }_{{\rm{ex}}}={\int }_{V}A{(\nabla {\bf{m}})}^{2}dV,$$

where ${\bf{m}}({\bf{r}})={\bf{M}}({\bf{r}})/{M}_{s}$ denotes the unit magnetization vector with *M*_*s*_ the saturation magnetization, **H**_0_ is the (constant) applied magnetic field, ${{\bf{H}}}_{D}({\bf{r}})$ is the magnetostatic field, ${K}_{u}$ is the (first-order) uniaxial anisotropy constant, **u** is a unit vector indicating the anisotropy-axis direction, *A* is the exchange-stiffness constant, ${\mu }_{0}$ denotes the permeability of free space, and the integrals are taken over the volume of the nanorod.

The nanorod is represented as a cylinder with a fixed length of $L=500\,{\rm{nm}}$, but with different diameters *D* (30, 60, and 90 nm); these dimensions are typical of real materials^12–17^. The magnetic materials parameters were set to the ones corresponding to bulk hcp Co^55^: ${M}_{s}=1.44\times {10}^{6}\,{\rm{A}}/{\rm{m}}$, ${K}_{u}=4.5\times {10}^{5}\,{\rm{J}}/{{\rm{m}}}^{{\rm{3}}}$, and $A=2.8\times {10}^{-11}\,{\rm{J}}/{\rm{m}}$; these values result in a magnetostatic exchange length of ${l}_{{\rm{ex}}}=\sqrt{2A/({\mu }_{0}{M}_{s}^{2})}=4.6\,{\rm{nm}}$ and in a domain-wall parameter of ${l}_{K}=\sqrt{A/{K}_{u}}=7.9\,{\rm{nm}}$. Correspondingly, a cubic cell with a side length of 2 nm was used in order to discretize the nanorod. The ${K}_{u}$-axis orientation with respect to the nanorods’s long axis was chosen in-plane along the nanorod’s diameter (${\bf{u}}=\mathrm{\{0,0,}\pm \mathrm{1\}}$ for ${{\bf{k}}}_{0}\perp {{\bf{H}}}_{0}$ and ${\bf{u}}=\{\pm \mathrm{1,0,0\}}$ for ${{\bf{k}}}_{0}\,\parallel \,{{\bf{H}}}_{0}$), in agreement with experimental observations which report a strong crystal anisotropy with an easy axis oriented nearly perpendicular to the axis of the wire (e.g., ref.^56^). The hysteresis loops were calculated as follows: the magnetic system was initially completely saturated by aligning the magnetic moments of all the computational cells along the *z*-axis. Then, the applied field was reduced in steps of 5 mT and the equilibrium magnetization state at each *H*_0_ (and therefore the static spin configuration) was found by two equivalent methods; we employed both the “Relax” and “Minimize” functions of Mumax3, where the former solves the Landau-Lifshitz-Gilbert equation without the precessional term and the latter uses the conjugate-gradient method to find the configuration of minimum energy. The translational invariance of the grid obtained with the finite-difference method enables the usage of the Fast Fourier transformation technique for the computation of the Fourier components of the magnetization. We have used the FFTW library^57^ to compute and analyze the Fourier components of our nanoscopic magnetic configurations. Thermal fluctuations were not taken into account in the simulations.

## Electronic supplementary material

Supplementary material
