# Publisher Correction: Small-angle neutron scattering modeling of spin disorder in nanoparticles

**DOI:** 10.1038/s41598-018-24855-5

**Published:** 2018-05-01

**Authors:** Laura G. Vivas, Rocio Yanes, Andreas Michels

**Affiliations:** 1 Physics and Materials Science Research Unit, University of Luxembourg, 162A avenue de la Faiencerie, Luxembourg, L-1511 Luxembourg; 2 Department of Applied Physics, University of Salamanca, Plaza de los Caidos, Salamanca, 37008 Spain

Correction to: *Scientific Reports* 10.1038/s41598-017-13457-2, published online 12 October 2017

In the PDF version of this Article, Equation 6 is formatted incorrectly. The correct Equation 6 appears below.

$$\begin{array}{rcl}{ {\mathcal E} }_{z} & = & -{\mu }_{0}{\int }_{v}{\bf{M}}\cdot {{\bf{H}}}_{{\rm{0}}}dV,\\ { {\mathcal E} }_{D} & = & -\frac{1}{2}{\mu }_{0}{\int }_{v}{\bf{M}}\cdot {{\bf{H}}}_{{D}}dV,\\ { {\mathcal E} }_{{\rm{ani}}} & = & -{\int }_{v}{{K}}_{{u}}{({\bf{m}}\cdot {\bf{u}})}^{2}dV,\\ { {\mathcal E} }_{{\rm{ex}}} & = & {\int }_{v}{A}{(\nabla {\bf{m}})}^{2}dV,\end{array}$$

