# Supplementary material for: Small-angle neutron scattering modeling of spin disorder in nanoparticles
Source: Sci Rep. 2017 Oct 12;7:13060. doi: 10.1038/s41598-017-13457-2 (PMC5638870; doi:10.1038/s41598-017-13457-2)
Supplement: Supplementary file 1 — Supplementary material [file 41598_2017_13457_MOESM1_ESM.pdf]

Supplemental Material

“Small-angle neutron scattering modeling of spin disorder in nanoparticles”

Laura G. Vivas,[1] Rocio Yanes,[2] Andreas Michels[1]

[1]*Physics and Materials Science Research Unit, University of Luxembourg, 162A avenue de la Faiencerie, L-1511 Luxembourg, Grand Duchy of Luxembourg*

[2]*Department of Applied Physics, University of Salamanca, Salamanca, 37008, Spain*

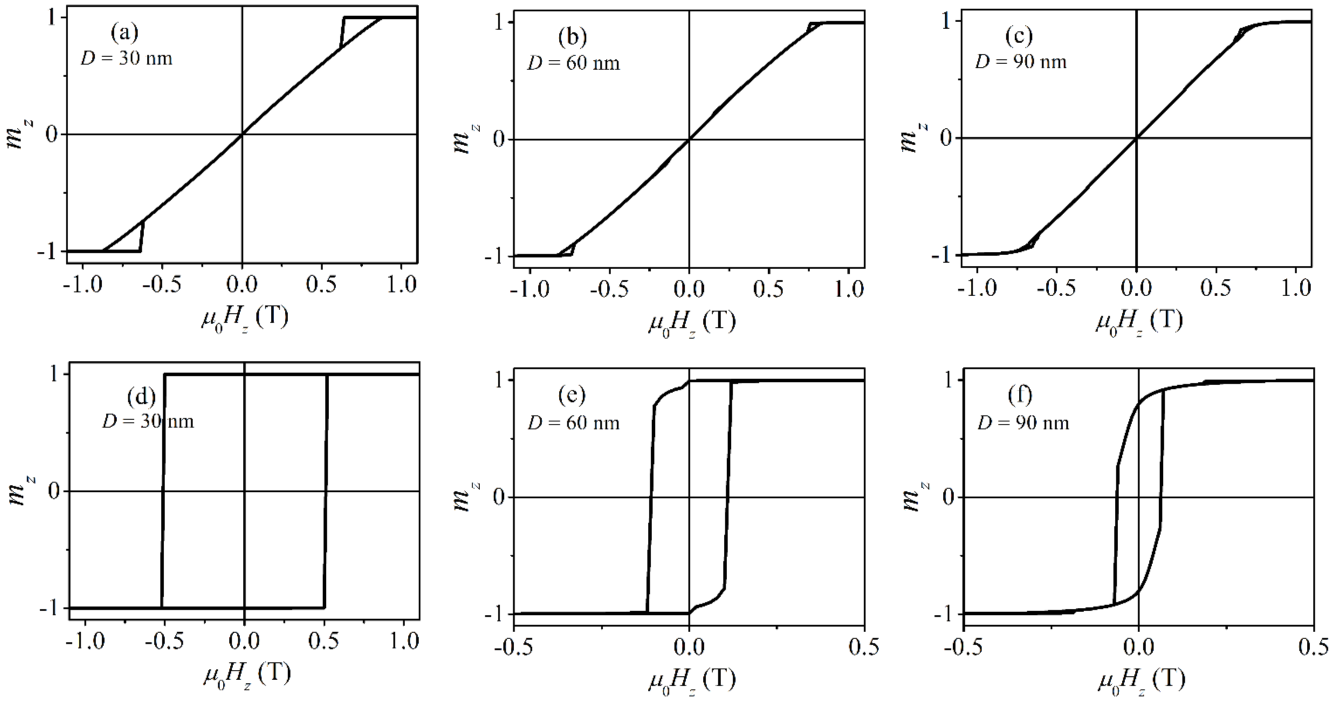

Figure 1: Normalized magnetization curves of a cobalt nanorod with zero magnetocrystalline anisotropy (K_u = 0). The length of the nanorod is L = 500 nm and the diameter D varies from 30-90 nm (see insets). (a)-(c): applied field $\mathbf{H}_0$ perpendicular to the wire axis; (d)-(f): $\mathbf{H}_0$ parallel to the wire axis.

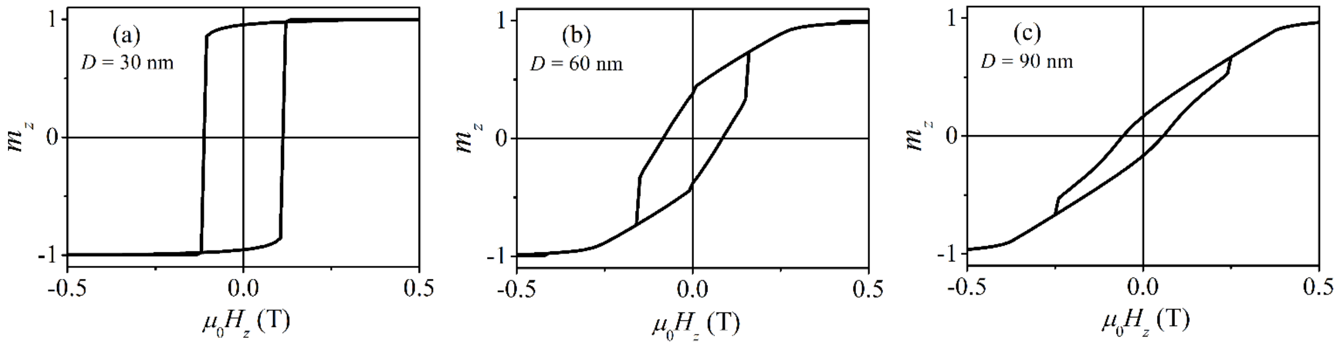

Figure 2: Magnetization curves of a cobalt nanorod (L = 500 nm; D = 30-90 nm). $\mathbf{H}_0$ is applied parallel to the wire axis. The uniaxial magnetocrystalline anisotropy axis ($K_u = 4.5 \times 10^5$ J/m^3) is perpendicular to the nanorod axis. Compare to Fig. 3 in the paper, which displays the results for $\mathbf{H}_0 \perp$ wire axis.

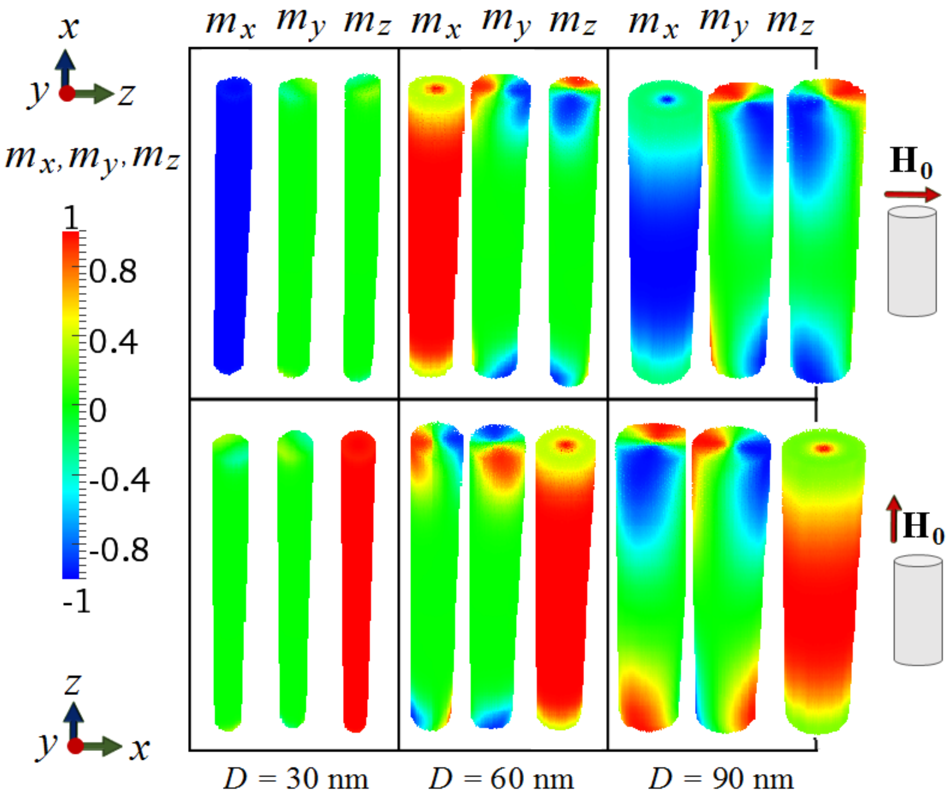

Figure 3: Computed spin structures of a cobalt nanorod in the remanent state for $K_u = 0$ and for different diameters D (L = 500 nm). Upper row: $\mathbf{H}_0$ perpendicular to the wire axis; lower row: $\mathbf{H}_0$ parallel to the nanorod axis.

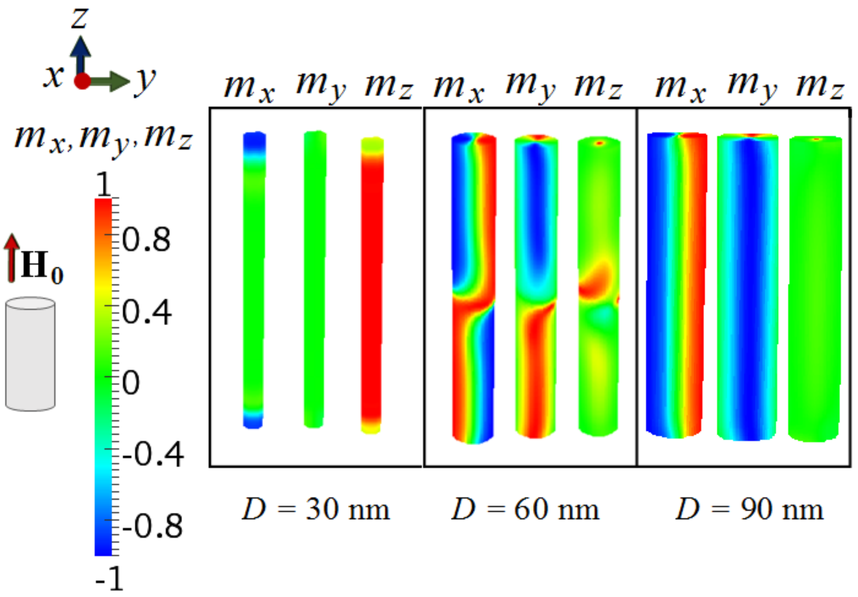

Figure 4: Computed spin structures of a cobalt nanorod in the remanent state and for different diameters D (L = 500 nm). $\mathbf{H}_0$ is applied parallel to the wire axis. The uniaxial magnetocrystalline anisotropy axis ($K_u = 4.5 \times 10^5$ J/m^3) is perpendicular to the nanorod axis. Compare to the insets in Fig. 3 in the paper, which show the results for $\mathbf{H}_0 \perp$ wire axis.

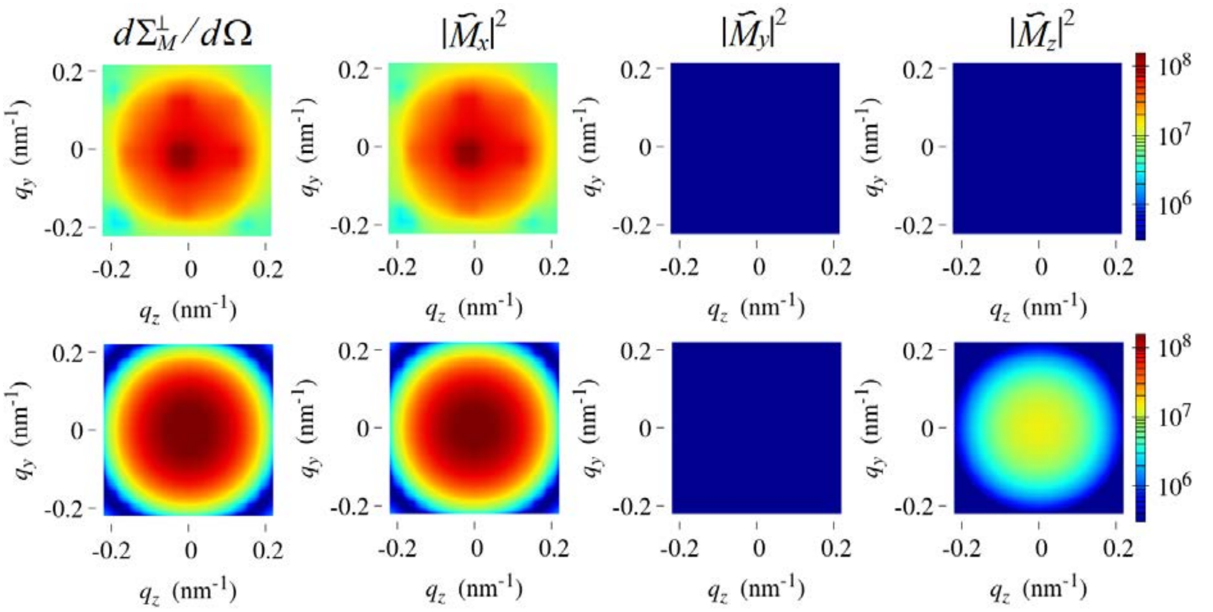

Figure 5: Computed perpendicular magnetic SANS cross section and Fourier coefficients of the magnetization in the remanent state for a cobalt nanorod (L = 500 nm; D = 30 nm) without (upper row) and with uniaxial anisotropy (lower row). The images represent projections of the respective function into the plane of the 2D detector (q_x = 0). Red color corresponds to high intensity and blue color to low intensity (logarithmic color scale).

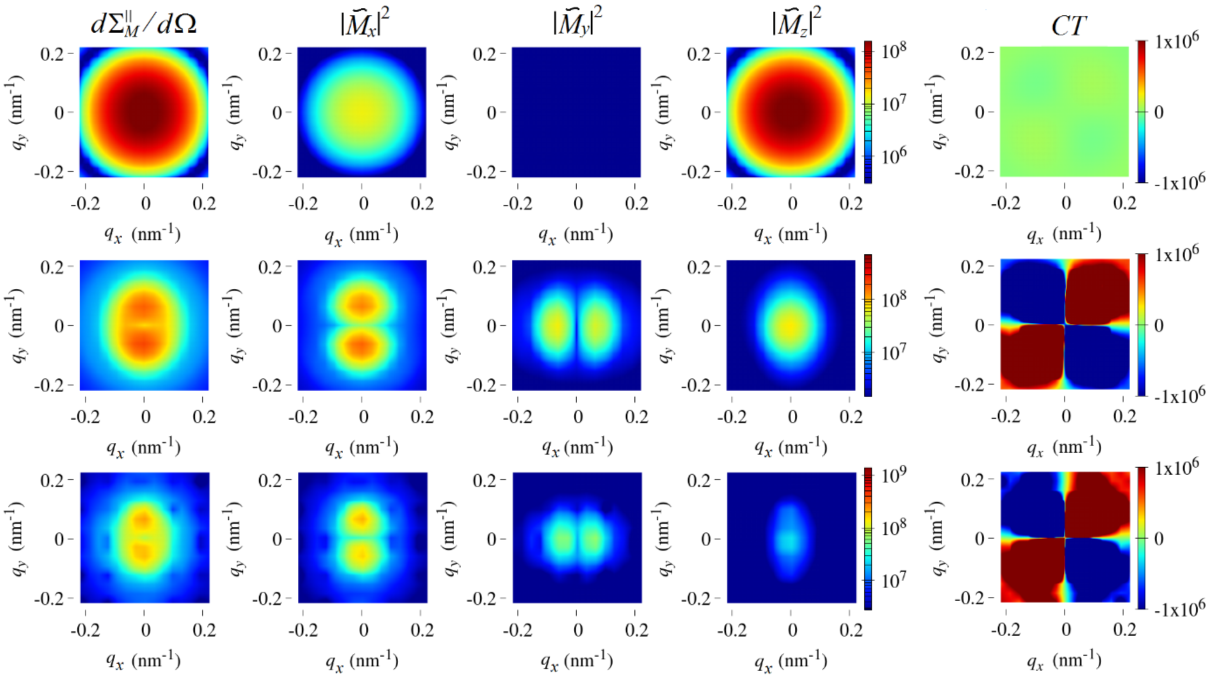

Figure 6: Total magnetic SANS cross section and Fourier components of the magnetization in the remanent state ($\mathbf{k}_0 \mid\mid \mathbf{H}_0$). The uniaxial magnetocrystalline anisotropy axis (K_u = 4.5 × 10^5 J/m^3) is perpendicular to the nanorod axis. The images represent projections of the respective function into the plane of the 2D detector (q_z = 0). $\mathbf{H}_0$ is normal on the plane. D = 30 nm (upper row); D = 60 nm (middle row); D = 90 nm (lower row). Compare to Fig. 4 in the paper, which show the results for $\mathbf{k}_0 \perp \mathbf{H}_0$.
